# Intelligibility of locally time-reversed speech: A multilingual comparison

**DOI:** 10.1038/s41598-017-01831-z

**Published:** 2017-05-11

**Authors:** Kazuo Ueda, Yoshitaka Nakajima, Wolfgang Ellermeier, Florian Kattner

**Affiliations:** 1 Department of Human Science/Research Center for Applied Perceptual Science, Kyushu University, 4-9-1 Shiobaru, Minami-ku, Fukuoka 815-8540 Japan; 2 Institut für Psychologie, Technische Universität Darmstadt, Alexanderstraße 10, D-64283 Darmstadt, Germany

## Abstract

A set of experiments was performed to make a cross-language comparison of intelligibility of locally time-reversed speech, employing a total of 117 native listeners of English, German, Japanese, and Mandarin Chinese. The experiments enabled to examine whether the languages of three types of timing—stress-, syllable-, and mora-timed languages—exhibit different trends in intelligibility, depending on the duration of the segments that were temporally reversed. The results showed a strikingly similar trend across languages, especially when the time axis of segment duration was normalised with respect to the deviation of a talker’s speech rate from the average in each language. This similarity is somewhat surprising given the systematic differences in vocalic proportions characterising the languages studied which had been shown in previous research and were largely replicated with the present speech material. These findings suggest that a universal temporal window shorter than 20–40 ms plays a crucial role in perceiving locally time-reversed speech by working as a buffer in which temporal reorganisation can take place with regard to lexical and semantic processing.

## Introduction

Speech reversal^1, 2^ is a simple but effective technique in investigating speech perception or speech-related effects. It produces pairs of speech-based stimuli that are matched in their long-term spectra, but yield opposite results regarding intelligibility: Playing a recorded spoken sentence backwards makes it totally unintelligible^3^. This technique has been applied to both behavioural^4, 5^ and neurophysiological^6^ investigations. It is especially convenient when a researcher would like to generate unintelligible stimuli, maintaining the long-term spectra and the range of spectral change. A surprising phenomenon involving reversed speech occurs when the disruptive effects of irrelevant speech on visual short-term memory are studied: Nearly the same amount of deterioration in performance is observed when presenting either intelligible speech or unintelligible reversed speech, even though the participants are instructed to ignore the sound^4, 7^. The time-reversing technique is, thus, very useful, but it is impossible to vary intelligibility gradually. If a spoken sentence is cut into very short (20 or 40 ms), successive segments, and each segment is reversed in time, listeners, in contrast, can report the sentence correctly. On the other hand, intelligibility declines when the segment duration is prolonged^8–15^ (Supplementary Audio S1 and Fig. S1). Locally time-reversed speech is crucial to address temporal aspects^16^ of speech perception.

Reversing speech samples locally in time is a technique first employed by Steffen and Werani^8^ in 1994 to the best of our knowledge. Each of their speech materials—nonsense sentences in German—was divided into two parts: the part below 300 Hz and the part above 300 Hz. The part below 300 Hz was preserved, while the part above 300 Hz was divided into successive temporal segments of 20–70 ms reversed in time. The resulting locally reversed speech sound were initially presented at segment durations of 70 ms, and the segment duration was then gradually shortened. For each segment duration, they counted the number of participants who correctly wrote down the whole sentence. The number of participants with perfect intelligibility increased as the duration of a segment decreased. The authors concluded that the majority of their participants understood the sentence perfectly with less than 40 ms of segment duration.

Saberi and Perrott^9^, seemingly unaware of the work by Steffen and Werani, published a study on locally time-reversed speech as a brief communication in *Nature*. They reversed each 20–300-ms segment of speech without any prior filtering or smoothing. All stimuli were generated from a single sentence. Seven participants were asked to rate intelligibility of each stimulus subjectively.

Later investigators of locally time-reversed speech employed a more objective measure of intelligibility—word intelligibility. Greenberg and Arai^10, 11^ presented 130 sentences to 27 participants, each spoken by a different talker including both genders and varying in American dialect regions, chronological ages, and voice quality^17^. Meunier *et al*.^12^ employed 28 French participants to make a comparison between the intelligibility curves of English and French—the English curve was taken from Greenberg and Arai. Kiss *et al*.^13^ focused on performance difference between German native and non-native listeners, 10 for each group; the non-native listeners performed significantly worse, compared to the native listeners. A wide range of speech rates—from the slowest rate of 2.5 syllables per second to the fastest rate of 10.0—was employed in the investigation by Stilp *et al*.^14^. This manipulation predictably shifted the intelligibility curves towards shorter segment durations. Remez *et al*.^15^ provided some evidence that subjective ratings could be a source of bias to make an intelligibility curve shallower than a curve obtained with a more objective method, along with presenting much deteriorated performance with locally time-reversed sine-wave speech, in which spectral information was greatly reduced. These results suggest that familiarity with a specific language, speech rates, and experimental methods are possible sources of variability, with regard to the intelligibility of natural speech that is segmented and reversed in time. When these variables were negligible, i.e., the original natural speech was produced at a moderate rate, and intelligibility was objectively measured with native listeners, the curves exhibited a rather similar trend; the 50%-intelligibility points fell in the range of 60–80 ms (Supplementary Fig. S2).

Thus, the effect of local time-reversal was confirmed with American English^9–11, 14, 15^, French^12^, and German^8, 13^, but their experimental protocols differed from study to study. For example, some authors presented a stimulus in a trial for a fixed number of times^13–15^, ranging from once to five times, whereas other researchers^10–12^ let their participants listen ad libitum up to four times on each trial. Some experimenters presented their stimuli in random order^10–12, 14^, whereas others presented them in a systematic order^13^. Those differences in experimental procedures might lead to some variations in the results. Although the inconsistencies may look subtle, they can be critical when considering brain mechanism.

A multi-time resolution model proposed by Poeppel and colleagues^18–23^ assumes that the auditory input has a bilaterally symmetric neural representation at an initial stage, and that subsequent processes include two different types of windows: a short temporal integration window that spans ~20–40 ms and a long temporal integration window of ~150–250 ms. The short integral window (or a sampling network) is a part of a mechanism that extracts speech information based on rapid spectral changes, i.e., consonants, whereas the long integral window has an advantage in extracting vowels. Lexical processes also interact with those two types of mechanism. Because the segment durations used in studies of time-reversed speech extend across both types of temporal windows, it is necessary to clarify whether some variations observed in the previous results were caused by language difference or artefacts.

Moreover, the different types of timing in languages have not yet been explored in previous investigations. It is common to categorise spoken languages into three types of timing, that is, stress-, syllable-, and mora-timed languages^24, 25^ (a *mora* is a syllable-like unit, but it is based on a regular time interval). Ramus *et al*.^24^ proposed that those three types of timing could be identified by acoustical measurements. They identified three types of languages in terms of the proportions of vocalic intervals in a sentence and standard deviations of consonantal intervals. The stress-timed languages in their analysis, i.e., English, Polish, and Dutch, were characterised by smallest proportions of vocalic intervals, the syllable-timed languages that they analysed, i.e., Spanish, French, Italian, and Catalan, by moderate proportions, and the mora-timed language, i.e., Japanese, by the largest proportion.

It is conceivable that those differences in timing affect the intelligibility of locally time-reversed speech. For example, *ojisan* (/o-ji-sa-N/, a middle-aged man or an uncle) has four morae, and $oj\bar{i}san$ (/o-ji-i-sa-N/, an old man or a grandfather) has five morae in Japanese. The second vowel in both words is the same, i.e., /i/, however, the length of it in the latter word is doubled. The difference might sound subtle for those who are unfamiliar with Japanese^26^, but actually it makes a semantic difference. That is, the feature in Japanese that just prolonging vowel duration by one mora can create a different word certainly contributes to relatively longer vocalic portions compared to other languages. This feature of Japanese may result in maintaining higher intelligibility in perceiving locally time-reversed speech than the other languages, because vowels are possibly less vulnerable when they are locally time-reversed than consonants that are transient in nature. If it holds, a similar but weaker trend may be found in a syllable-timed language, e.g., Mandarin Chinese; Mandarin Chinese is thought to be a prototypical syllable-timed language by phoneticians^25^.

Therefore, the purpose of the present investigation was to examine the intelligibility of spoken sentences in American English, German, Japanese, and Mandarin Chinese as the duration of locally time-reversed segments changed. American English and German were considered as a kind of control, representing stress-timed languages, Mandarin Chinese as an example of a syllable-timed language, and Japanese as a mora-timed language. Our initial hypothesis was that the types of speech rhythm affect intelligibility of locally time-reversed speech; more specifically, stress-timed languages, i.e., American English and German, are most vulnerable to local time-reversal, a syllable-timed language, i.e., Mandarin Chinese, falls in-between, and a mora-timed language, i.e., Japanese, is least vulnerable. The alternative hypothesis was that intelligibility is entirely determined by the segment length of the local time-reversals, not by language rhythm types. In that case, a universal mechanism that retrieves plausible speech from locally time-reversed speech determines performance. To make a fair comparison, syllable intelligibility rather than word intelligibility was measured in American English, German, and Mandarin Chinese, while mora intelligibility was measured in Japanese, under the same experimental procedure.

## Results

**Figure 1:**
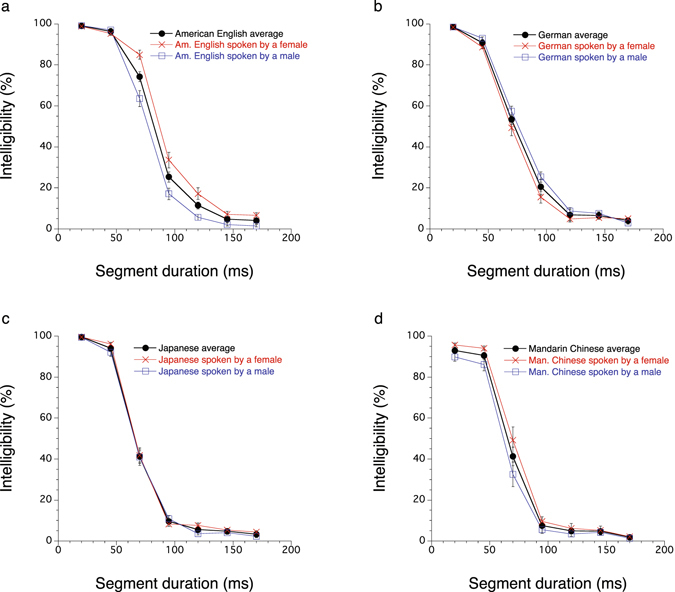
Percentage of syllable or mora intelligibility in each language as a function of reversed-speech segment duration. All the data except in (**d**) were based on the test results with 35 sentences and 28 participants (a half of the participants heard sentences spoken by a female, and the other half heard a male). (**a**) American English, (**b**) German, (**c**) Japanese, and (**d**) Mandarin Chinese. Chinese results (**d**) obtained with 18 sentences and 27 participants (14 heard a female talker, 13 heard a male). Error bars represent s.e.m.

Figure 1 (see also Supplementary Table S1) represents the results for each language. The curves in the figure show similar trends across different languages. Separate analysis of variance (ANOVA) on arcsine-transformed intelligibility, specifying talkers and participants as random effects, revealed that the main effect of the segment duration was significant in all languages [American English: *F*(6, 946) = 864.52, *p* < 0.01; German: *F*(6, 946) = 613.29, *p* < 0.01; Japanese: *F*(6, 946) = 1170.59, *p* < 0.01; Mandarin Chinese: *F*(6, 465) = 361.16, *p* < 0.01]. Utterance duration differences between the female and male talkers in each language (summarised in Supplementary Table S2) were examined with paired *t*-tests (two-tailed). The analysis revealed that the female talker of American English took significantly longer than the male talker [0.64 s on average, *SE* = 0.047; *t*(34) = 13.72, *p* < 0.01], and that the female talker in German took significantly less time than the male talker [−0.06 s on average, *SE* = 0.023; *t*(34) = −2.73, *p* = 0.01]. No significant duration difference was observed in Japanese and Mandarin Chinese [Japanese: 0.09 s on average, *SE* = 0.045; *t*(34) = 1.89, *p* = 0.07; Mandarin Chinese: −0.07 s on average, *SE* = 0.058; *t*(17) = −1.24, *p* = 0.23]. A mixed ANOVA applied to the data pooled across languages revealed that the main effects of segment duration [*F*(6, 3363) = 2521.65, *p* < 0.01] and the interaction effect between segment duration and language [*F*(18, 3346) = 15.33, *p* < 0.01] were significant. The main effect of language was not significant [*F*(3, 4) = 2.95, *p* = 0.16]. A post hoc analysis was performed as to the difference of intelligibility (arcsine transformed) between the 45- and 70-ms segment-duration conditions. A paired *t*-tests (two-tailed) showed a statistically significant difference between these two conditions [*t*(486) = −23.13, *p* < 0.01].

**Figure 2:**
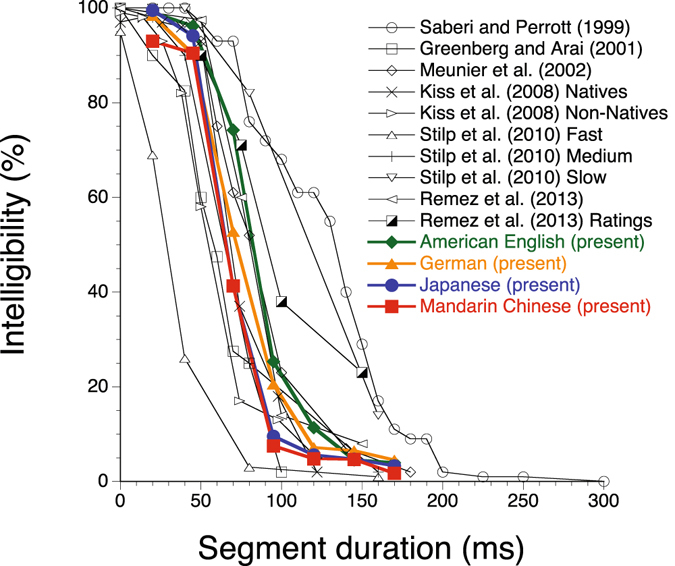
Summarised results of the previous and present investigations. Data collected with locally time-reversing natural speech materials spoken at a moderate speech rate, employing an objective method and native listeners, exhibited a similar trend.

**Figure 3:**
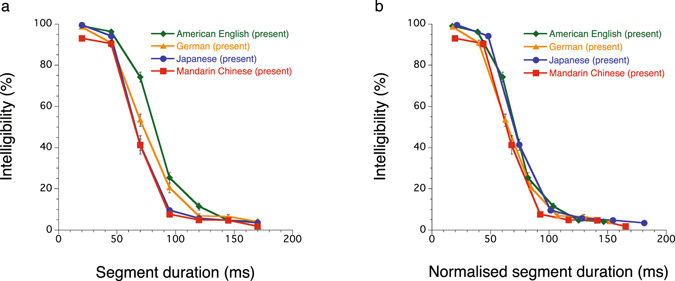
Normalising segment duration with speech rates. (**a**) Original intelligibility curves obtained in the present investigation, (**b**) the segment duration was normalised to cancel out the speech rate deviations of the individual talkers from the average in each language, by dividing the segment duration by a ratio of an average sentence duration as to a given talker or a pair of talkers to the average sentence duration over all the talkers in the database in each language (see Supplementary Table S2). Error bars represent s.e.m.

Figure 2 shows a summary of the results available to date. The curves of the present results and the majority of the previous studies largely agree; the exceptions are Saberi and Perrott^9^, the slow and fast speech-rate conditions in Stilp *et al*.^14^, and the rating condition in Remez *et al*.^15^. It is possible that a slow speech rate, a subjective rating of intelligibility that is not comparable to objective performance data per se, or both affected the results of Saberi and Perrott. As to the present results, the averages of the 50%-intelligibility points computed per language fell into the 16-ms range from 66 to 82 ms (see also Fig. 3a).

In addition, percentage of tone errors—i.e., errors in tone sign responses in Mandarin Chinese—was examined. Syllables that were correct in Pinyin spellings but incorrect only in tone signs were counted. The error percentage was calculated from a proportion of tone errors to a number of responded syllables in a sentence (Supplementary Table S3). A Welch test revealed a significant effect of segment durations on the arcsine transformed percentage of tone errors [*F*(6, 166.97) = 5.58, *p* < 0.01], i.e., the transformed percentage of tone errors increases with lengthening the segment durations.

So far, no direct—within participants—comparison of the intelligibility of locally time-reversed speech between native vs. non-native languages has been performed. Therefore, an additional experiment was run, where two Mandarin Chinese native listeners with normal hearing (ages, 23 years in both cases), having studied Japanese for 1 or 4 years, heard both Mandarin Chinese and Japanese. The intelligibility curves for Japanese generally fell well below those for Mandarin Chinese (Supplementary Fig. S3; see also Supplementary Table S4). A fixed ANOVA with the arcsine transformed data showed significant main effects of segment duration [*F*(6, 126) = 12.30, *p* < 0.01] and language [*F*(1, 126) = 59.80, *p* < 0.01], and a significant interaction effect between segment duration and language [*F*(6, 126) = 3.23, *p* < 0.01], which revealed a clear effect of participants’ language familiarity.

## Discussion

Speech rate is an important factor that may shift an intelligibility curve drastically, as Stilp *et al*.^14^ showed (Fig. 2). Making a speech rate faster has an effect on intelligibility comparable to making a segment duration longer, putting more events into each reversed segment. Thus, we attempted to normalise the present data with respect to the varying speech rates of different talkers (see Methods, Normalising speech rate; Supplementary Table S2). Figure 3 shows how such normalisation worked; the curves look even more similar to each other with the normalisation (Fig. 3b). Besides, the range of 50%-intelligibility points was reduced to 8 ms, from 63 to 71 ms, suggesting a universal perceptual mechanism in all three types of languages.

One might speculate that the tone errors in Mandarin Chinese are caused by local time-reversal, because reversal may distort perceived tones. If that happens, the percentages of tone errors should increase with lengthening segment durations; the statistical analysis supports the idea. Thus, the tone errors possibly lower the performance in Mandarin Chinese compared to the other non-tonal languages. In any case, it is confirmed that the errors are less than about 5% on average (Supplementary Table S3) and too small to alter our conclusions.

**Figure 4:**
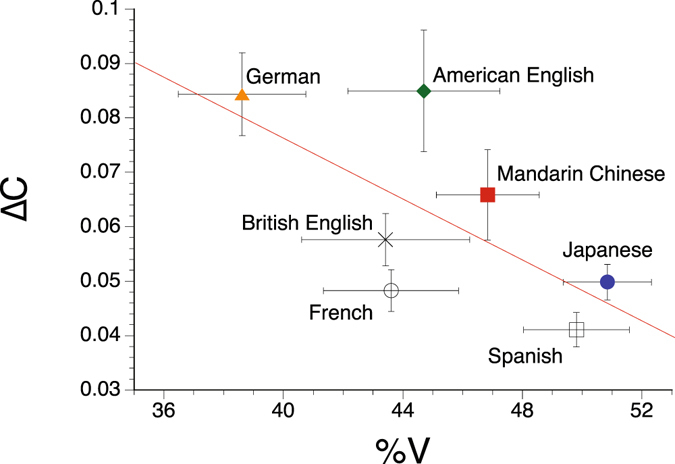
Replication of Ramus *et al*.^24^. The horizontal axis represents the percentage of vocalic intervals in a sentence. The vertical axis represents standard deviation of consonantal intervals in a sentence. Five sentences in American English, German, Japanese, and Mandarin Chinese were randomly sampled from 35 (in the case of Mandarin Chinese, 18) sentences used in the intelligibility measure. The analysis results of these languages are represented by coloured symbols. Other languages, i.e., British English, French, and Spanish, are for reference, based on five sentences randomly selected from the same database (represented in black symbols). Error bars represent s.e.m. The red line shows a regression line (Δ*C* = 0.1862 − 0.0027 × %*V*; *R* ^2^ = 0.43), on which the distance among languages can be determined by putting perpendicular lines from the points representing the languages to the scale. The positions of the languages on the scale are ordered according to the rhythm categories with no exception: from left to right, German, American English, and British English can be grouped together as stress-timed languages, French, Mandarin Chinese, and Spanish as syllable-timed, and Japanese as mora-timed.

The observed discrepancies of intelligibility curves between different studies (see Fig. 2) thus do not seem to be due to differences in the timing of languages (Fig. 4), but they can be accounted for by between-talker differences in speech rate. The initial hypothesis claiming the effects of speech rhythm thus does not hold. The alternative hypothesis assuming a universal mechanism for retrieving plausible speech from locally time-reversed speech is supported.

The multi-time resolution model proposed by Poeppel and colleagues^18–23^ may fit into the above-mentioned universal mechanism. The present results showed that speech perception was almost intact as long as the reversal window was shorter than 45 ms (Fig. 3b). This fact appears to be in line with the concept of the short integral window in the model, because the model implies that if the extraction of consonants fails, intelligibility should drastically go down. If this holds, the intelligibility curves should be essentially independent of speech timing, which is largely influenced by the proportion between vocalic and consonantal intervals.

Seen in a broader context, the current investigation can be placed in the flow of several multilingual studies published recently^27–29^. Among them, it is especially worth noting that a study on the speech modulation spectrum demonstrates striking similarities among languages: Ding *et al*.^29^ show that the speech modulation spectrum is highly consistent across nine languages/dialects with a peak frequency at 4–5 Hz (~200 ms expressed in a period). This study implies a global window of ~200 ms for speech analysis, whereas our results provide a support for a local window of ~20–40 ms. Moreover, studies employing a behavioural approach^20, 23^ and event-related potentials (ERPs)^20^ with non-speech stimuli also provide support for the multi-time resolution model.

Therefore, the present results apparently suggest that a universal time-integrating mechanism works in speech perception across different types of languages: be they stress-timed, syllable-timed, or mora-timed. However, it should not be taken to be suggesting that the auditory system always integrates the temporal information within a short window below 40 ms. Instead, the window of 40 ms or less should be considered as a buffer, within which the temporal order of events can be reorganised according to lexical and semantic processing, because Kiss *et al*.^13^ showed statistically significant differences between native and non-native listeners with respect to the intelligibility of locally time-reversed speech—a difference of 14% in intelligibility was observed between the two groups at segment durations of 38 ms (Supplementary Fig. S2)—, because our Mandarin Chinese native listeners also exhibited deteriorated performance in their non-native language, i.e., Japanese (Supplementary Fig. S3 and Table S4), and because Ishida *et al*.^30^ found that words had clear advantage in intelligibility compared to pseudo-words even when the reversal segment durations were shorter than 40 ms. It is possible that the efficiency of those lexical and semantic processes may be reflected in the processing speed of the temporal reorganisation; the auditory system has to override local time-reversal within a limited time, accessing a mental lexicon, and evaluating semantics, while simultaneously accepting new input. These acrobatics should become even more challenging if the input is a pseudo- or a foreign word. The present investigation shows a possibility to use an objective measure of intelligibility on locally time-reversed speech as a universal index of neural processing speed across different languages.

## Methods

### Participants

Twenty-eight native speakers of Japanese, 10 females and 18 males (age, 21–23 years; median, 22 years), and 28 native speakers of Mandarin Chinese, 17 females and 11 males (age, 21–32 years; median, 24 years), participated in the experiments performed at Kyushu University, and 28 native speakers of German, 4 females and 24 males (age, 18–53 years; median, 22 years), and 28 native speakers of English, 9 females and 19 males (age, 20–59 years; median, 22 years), participated in the experiments performed at Technische Universität Darmstadt. All the participants passed a screening test with a clinical audiometer to ensure that they had normal hearing within the frequency range of 250–8,000 Hz^31^. They were divided into two groups, each of which was allotted to the stimuli produced by a male speaker or a female speaker. Informed consent was obtained from each participant prior to performing the experiments. The research was conducted with prior approval of the Ethics Committee of Kyushu University; all methods employed were in accordance with the guidelines provided by the German Psychological Association (DGPs) and the Japanese Psychological Association (JPA).

### Stimuli

Thirty-five sentences in American English, German, Japanese, and Mandarin Chinese spoken by a male and a female speaker, were extracted from NTT-AT Multilingual Speech Database 2002 (NTT-AT, Kawasaki, Japan; recorded with a 16-kHz sampling rate and 16-bit linear quantisation) with eliminating irrelevant blanks and noises. Each sentence was segmented with a fixed duration in each condition. Seven segment duration conditions, ranging from 20–170 ms at 25-ms intervals including 2.5-ms cosine ramps to fade in and fade out each segment, were constructed. Then, each segment was reversed in time and subsequently joined in the original order.

### Apparatus

Exactly the same way of stimulus presentation was employed in both experiment sites, i.e., Kyushu University and Technische Universität Darmstadt laboratories, using headphones of the same make [Beyerdynamic DT 990 (Beyerdynamic GmbH, Heilbronn, Germany)] in a soundproof booth [Kyushu: Music cabin SC3 (Takahashi Kensetsu, Kawasaki, Japan); Darmstadt: Industrial Acoustics Company (Niederkrüchten, Germany)]. The sound pressure levels of the stimuli at the headphones were adjusted to 74 dB SPL with a 1-kHz calibration tone, which was provided with the speech database, by using an artificial ear [Brüel & Kjær type 4153 (Brüel & Kjær Sound & Vibration Measurement A/S, Nærum, Denmark)], a condenser microphone (Brüel & Kjær type 4192), and a sound level meter (Brüel & Kjær type 2250). An optical interface [USB interface Roland UA-4FX (Roland Corp., Shizuoka, Japan)] and a headphone amplifier with a built-in DA converter [Audiotechnica AT-DHA 3000 (Audiotechnica, Machida, Japan)] were used at Kyushu University, and a DA converter [RME multiface II (Audio AG, Haimhausen, Germany)] and a headphone amplifier [Behringer Pro 8 (Behringer, Zhongshan, China)] were used at Technische Universität Darmstadt to drive the headphones.

### Procedure

A sequence of 35 trials for each participant consisted of five blocks of seven trials/sentences. In each block, the seven conditions (i.e. segment durations) were randomly permuted. The combination of a given sentence with a given segment duration as well as its position in a block were counterbalanced across participants.

Each sentence was presented on just a single trial to each participant. The stimuli were presented diotically to the participants through the headphones. Within each trial, the sentence was repeated three times with an inter-stimulus-interval of 1 s before a participant started to answer. The participant was instructed to write down what he/she heard without guessing. They were instructed to respond in alphabets in the American English and German conditions, in hiragana or katakana in the Japanese condition, and in Pinyin with tone signs in the Mandarin Chinese condition. When only parts of the sentence were understood, the participant was instructed to record the portions at their approximate locations on a scale representing the length of a sentence. The indices of intelligibility were a percentage of correctly reported syllables in American English, German, and Mandarin Chinese, and a percentage of correctly reported morae in Japanese. The number of correct responses was assessed by three independent scorers in American English and German, and by two independent scorers in Japanese and Mandarin Chinese. All the intelligibility data were arcsine-transformed before being submitted to statistical analysis.

The data obtained from one participant in Mandarin Chinese who heard sentences spoken by a male talker had to be totally discarded because, against the instructions, the participant completely omitted tone signs, making it impossible to score the responses in a fair way. It was also realised that 17 sentences out of 35 were unmatched between the two talkers in the Mandarin Chinese experiment. Therefore, only the data obtained from matched 18 sentences were kept, and the remainder of the Mandarin Chinese results was discarded. To check whether the exclusion introduced a flaw into the results, an additional experiment using a full set of 35 Chinese sentences—matched between the two talkers—with 6 Mandarin Chinese native participants with normal hearing (ages, 23–30 years; median, 26 years) was performed. The results are indicated in Supplementary Fig. S4 and in Supplementary Table S5, showing no serious discrepancy between the two sets of results. Thus, it looks like that the dataset with 18 sentences is fairly reliable.

### Normalising speech rates

The effect of speech rates on intelligibility of locally time-reversed speech among different talkers of different languages was compensated according to the following procedure. First, the durations of the sentences used in the present experiment were measured with the sentences spoken by as many talkers as possible in the database: 10 female and 10 male talkers in American English, and 5 female and 5 male talkers in German, Japanese, and Mandarin Chinese. Second, the ratio of the duration of the sentences spoken by a given talker (or a pair of talkers) whose speech materials were used in the experiment to the average duration of all talkers was calculated (Supplementary Table S2). Finally, the segmentation durations were divided by the ratio of each talker (or a pair of talkers) in each language, resulting in the normalised segment durations defining the abscissa in Fig. 3b.

### Replication of speech timing measure

The timing measure used by Ramus *et al*.^24^ was employed. Five sentences spoken by both a female and a male talker in American English, German, Japanese, and Mandarin Chinese were randomly selected from the 35 (in the case of Mandarin Chinese, 18) sentences that were used in the experiment. The random selection of five sentences was also done in British English, French, and Spanish in the same speech database. One of the authors (K.U.), who understands English, French, German, and Japanese, performed the measurements of vocalic and consonantal intervals.

## Electronic supplementary material

Supplementary Audio

Supplementary Information
