# Supplementary material for: Intelligibility of locally time-reversed speech: A multilingual comparison
Source: Sci Rep. 2017 May 11;7:1782. doi: 10.1038/s41598-017-01831-z (PMC5431844; doi:10.1038/s41598-017-01831-z)
Supplement: Supplementary file 2 — Supplementary Information [file 41598_2017_1831_MOESM2_ESM.pdf]

Supplementary Information
Intelligibility of locally time-reversed speech: A multilingual comparison

Kazuo Ueda[1*], **Yoshitaka Nakajima**[1*], **Wolfgang Ellermeier**[2*], **& Florian Kattner**[2*]

[1]Department of Human Science/Research Center for Applied Perceptual Science, Kyushu University, 4-9-1 Shiobaru, Minami-ku, Fukuoka 815-8540, Japan
[2]Institut für Psychologie, Technische Universität Darmstadt, Alexanderstr. 10, D-64283 Darmstadt, Germany
*email to K.U.: ueda@design.kyushu-u.ac.jp; email to Y.N.: nakajima@design.kyushu-u.ac.jp; email to W.E.: ellermeier@psychologie.tu-darmstadt.de; email to F.K.: kattner@psychologie.tu-darmstadt.de

Table of Contents

Supplementary Audio S1
Supplementary Figures S1–S4
Supplementary Tables S1–S5
References

Supplementary Audio

Supplementary Audio S1. An example of reversed and locally time-reversed speech. See detail in the caption of Supplementary Figure S1. The original speech sample was taken from NTT-AT Multi-lingual Speech Database 2002[1]. The use of the original and its modified versions were granted permission by NTT-AT.

Supplementary Figures

a

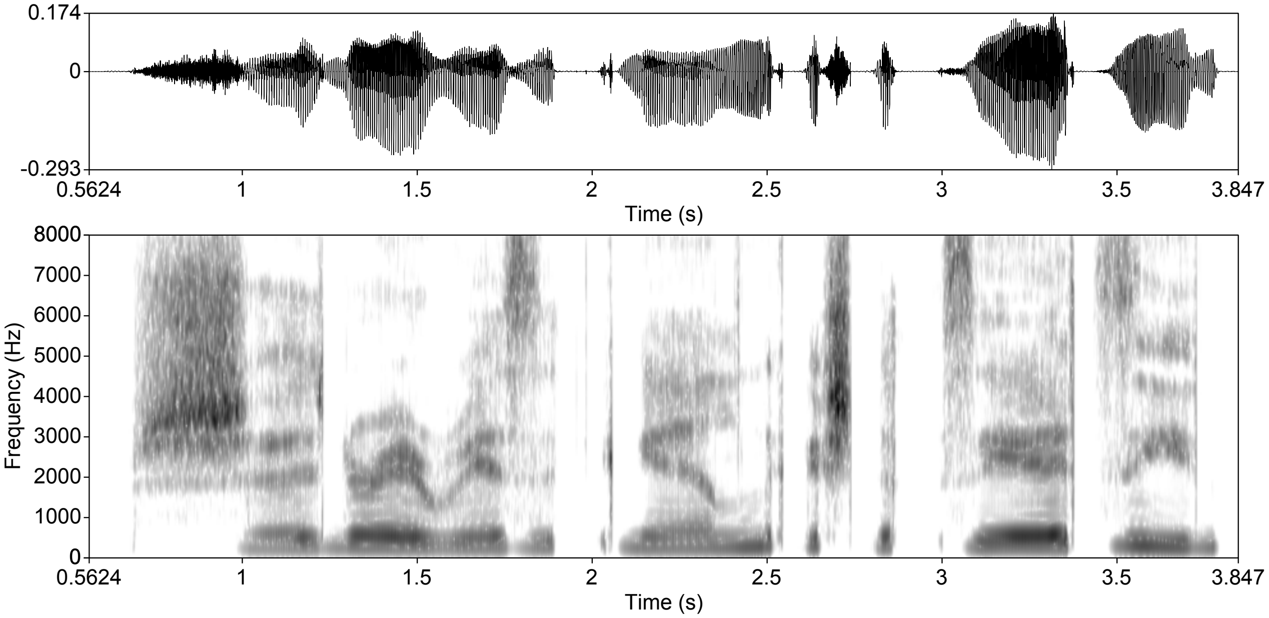

b

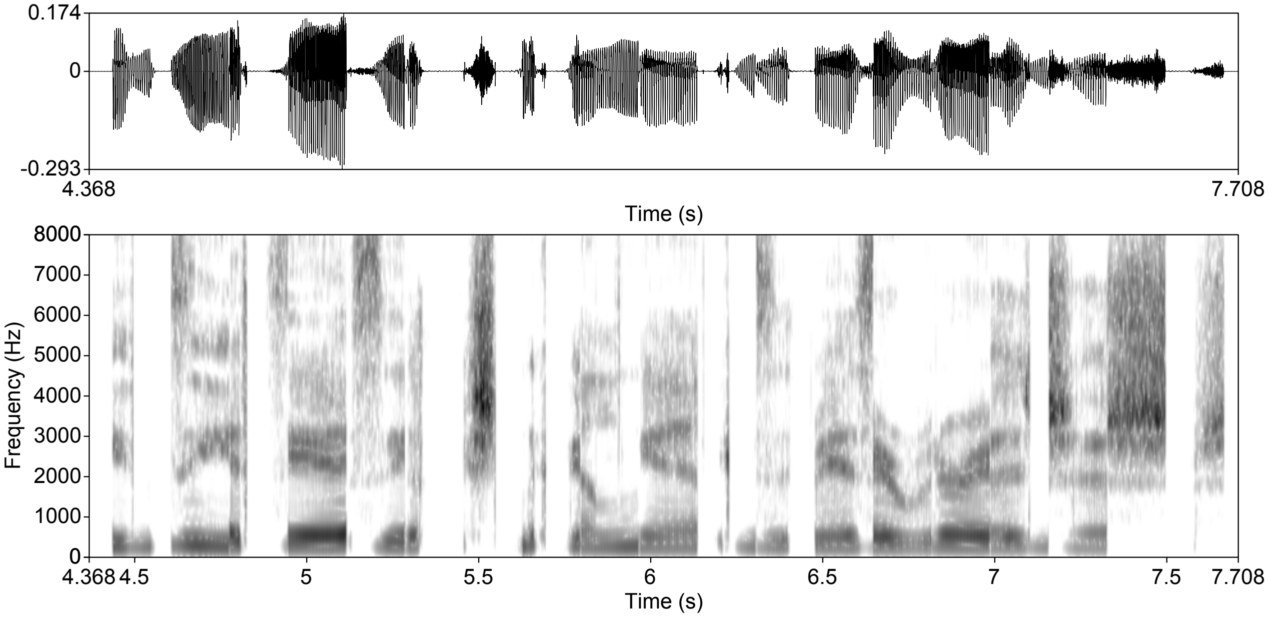

c

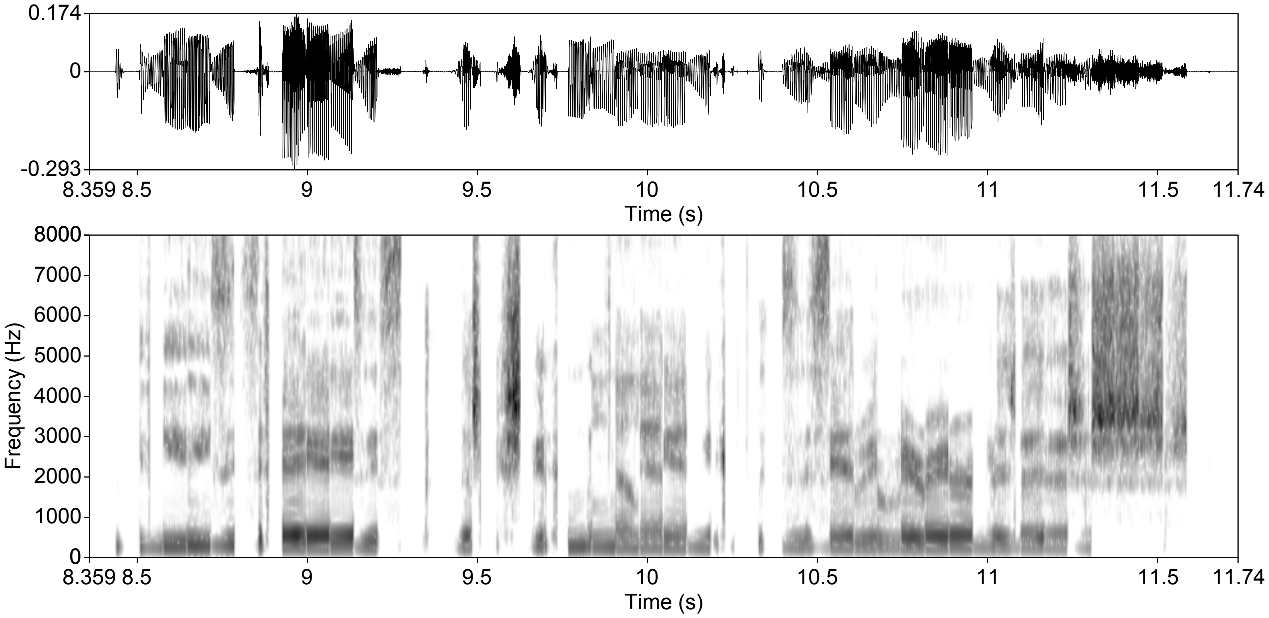
0.174
0
-0.293
8.359 8.5
9
9.5
10
10.5
11
11.5
11.74
Time (s)
8000
7000
6000
5000
4000
3000
2000
1000
0
Frequency (Hz)
8.359 8.5
9
9.5
10
10.5
11
11.5
11.74
Time (s)

d

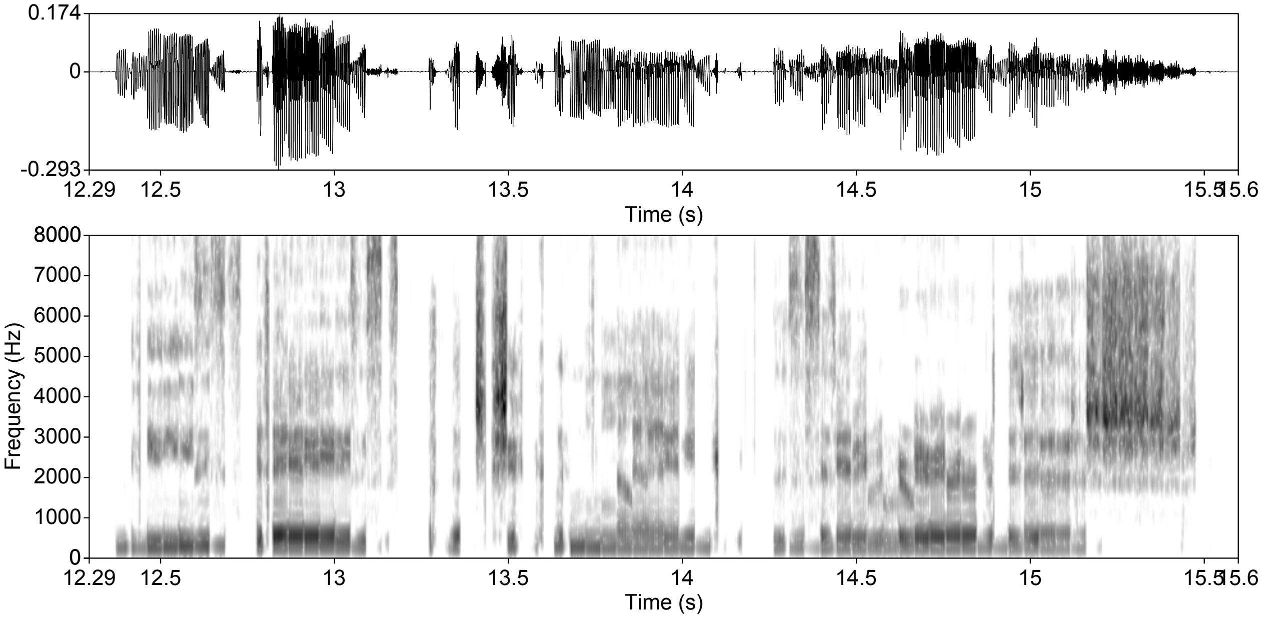
0.174
0
-0.293
12.29
12.5
13
13.5
14
14.5
15
15.5
15.6
Time (s)
8000
7000
6000
5000
4000
3000
2000
1000
0
Frequency (Hz)
12.29
12.5
13
13.5
14
14.5
15
15.5
15.6
Time (s)

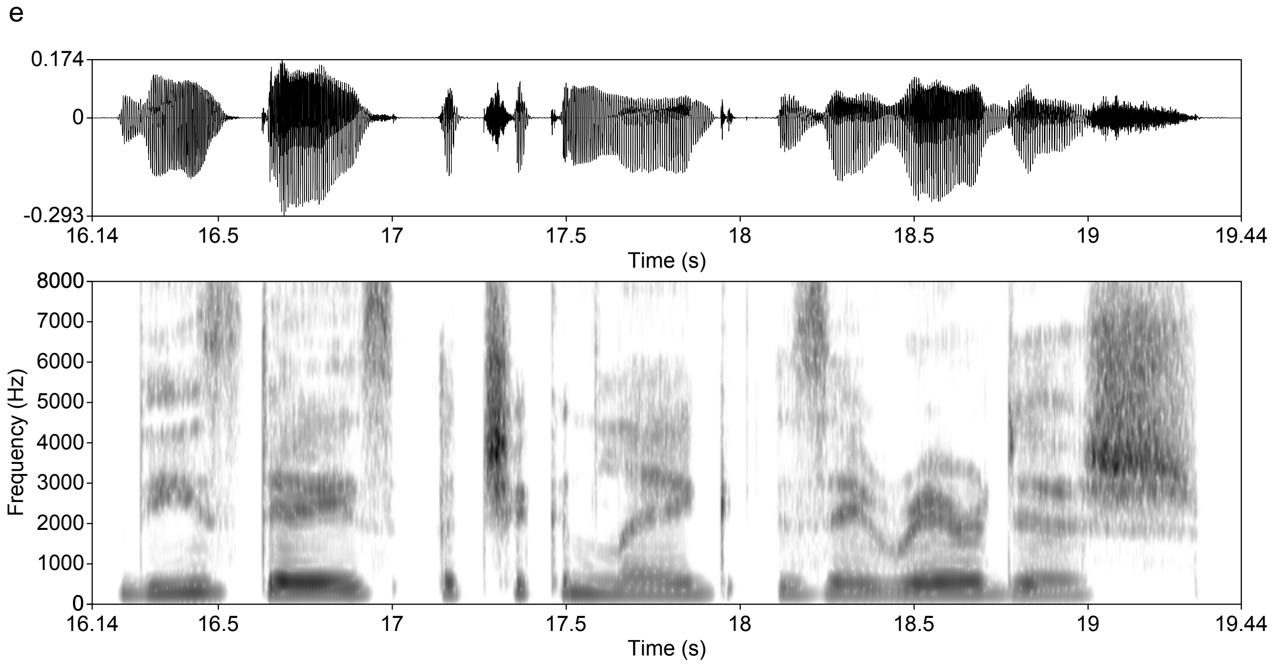

Supplementary Figure S1. The waveforms (upper panels) and spectrograms (lower panels) of Supplementary Audio S1. **a**, Reversed speech, **b-d**, locally time-reversed speech with segment duration of 170, 70, and 45 ms respectively, and **e**, the original spoken sentence in American English. The reversed speech, **a**, is a simply time-reversed version of the original, **e**. These two waveforms and spectrograms are reversed like a mirror image. The long-term spectra are the same, but **a** is completely unintelligible. The locally time-reversed versions, **b–d**, basically preserve the same long-term spectra as in the original, but their intelligibility depends on their segmentation durations: **b** is unintelligible, **c** is only partially intelligible, and **d** is mostly intelligible; nevertheless, the spectrograms show deteriorative effects of time reversal—e.g., intrusion of a silence/vowel in the middle of a consonantal burst and resulting fragmentation, unnatural movements of formant transitions and decay—even in the shortest segmentation duration condition, **d**. Our brain can overcome the distortion when a segmentation duration is short enough compared to the speed of articulatory movements of our familiar language. The original speech sample was taken from NTT-AT Multi-lingual Speech Database 2002[1]. The use of the original and its modified versions were granted permission by NTT-AT. These graphs were produced with Praat[2].

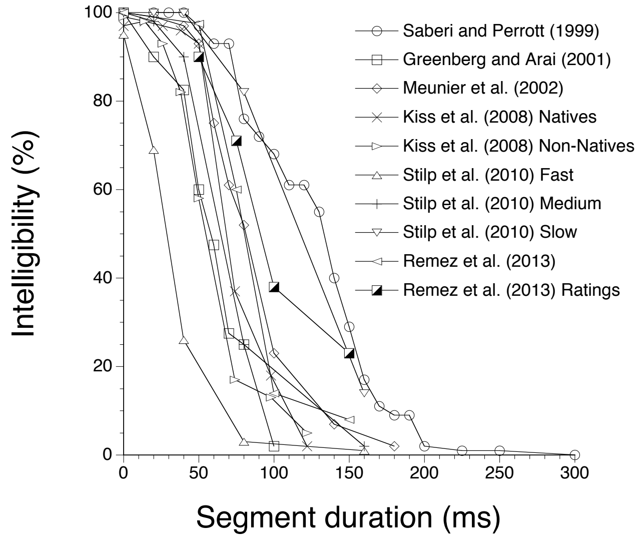

Supplementary Figure S2. Summary of previous intelligibility measures in locally time-reversed speech.

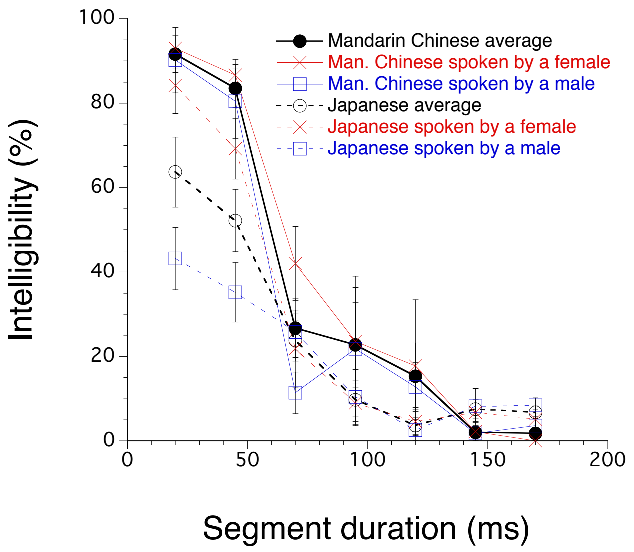

Supplementary Figure S3. Cross-language comparison with Mandarin Chinese and Japanese, heard by Mandarin Chinese native speakers. Two Mandarin Chinese native speakers with normal hearing heard both Mandarin Chinese and Japanese. A full set of 35 sentences matched between the two talkers in each language was employed. Each participant was assigned to either a female talker or a male talker in each language. The Mandarin Chinese data are also included in Supplementary Fig. S4. Error bars represent s.e.m.

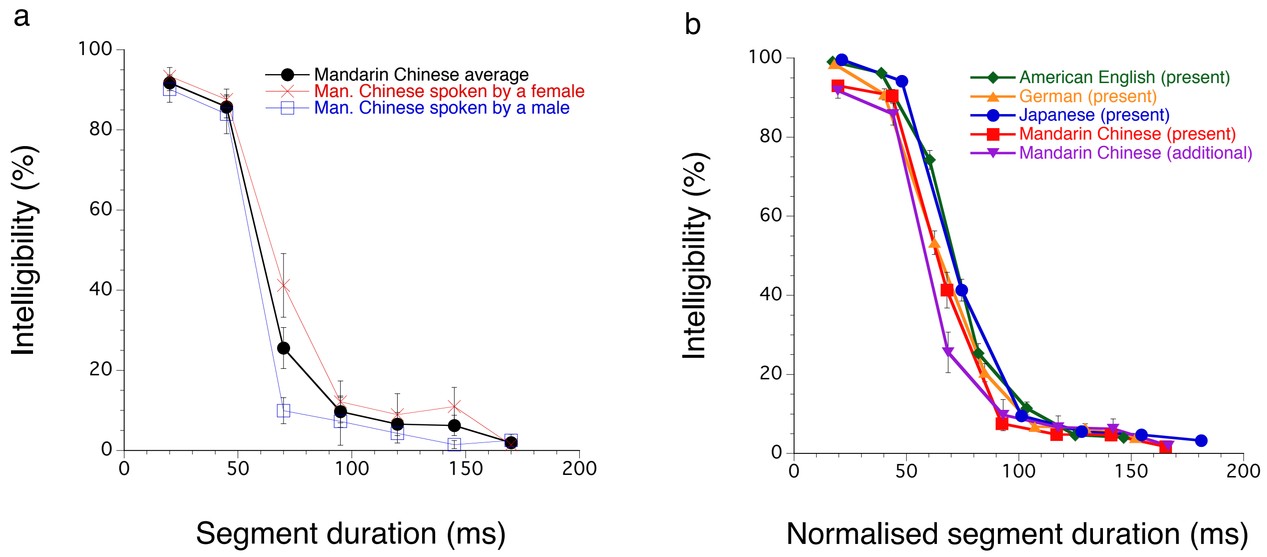

Supplementary Figure S4. Additional data in Mandarin Chinese. a, Mandarin Chinese results obtained with a full set of 35 sentences (matched between the two talkers) and 6 participants (3 for a female talker and 3 for a male), **b**, the averaged curve overlaid with Fig. 3b. Error bars represent s.e.m.

Supplementary Tables

Supplementary Table S1. Plotted data of Figures 1 and 3, i.e., percentage of syllable or mora intelligibility in each language as a function of reversed-speech segment duration. In Figure 3b, the segment duration was normalised according to ratios shown in Supplementary Table S2. Numbers in parentheses are s.e.m.

Language	Segment duration (ms)	Talker		Average
		Female	Male	
American English	20	98.8 (0.43)	99.2 (0.42)	99.0 (0.30)
	45	95.2 (0.98)	97.1 (0.83)	96.2 (0.64)
	70	84.9 (2.19)	63.6 (3.81)	74.2 (2.37)
	95	33.6 (3.78)	17.0 (2.90)	25.3 (2.48)
	120	17.2 (2.79)	5.6 (1.48)	11.4 (1.65)
	145	7.1 (1.52)	2.1 (0.73)	4.6 (0.86)
	170	6.5 (1.44)	1.5 (0.56)	4.0 (0.80)
German	20	98.4 (0.65)	98.5 (0.54)	98.4 (0.42)
	45	88.6 (2.31)	93.0 (1.63)	90.8 (1.42)
	70	49.3 (4.36)	57.4 (4.14)	53.3 (3.01)
	95	15.6 (3.04)	25.3 (3.28)	20.4 (2.27)
	120	4.9 (1.48)	8.7 (1.58)	6.8 (1.09)
	145	5.4 (1.40)	7.6 (1.63)	6.5 (1.08)
	170	5.0 (1.21)	2.9 (1.01)	4.0 (0.79)
Japanese	20	99.6 (0.27)	99.4 (0.20)	99.5 (0.19)
	45	96.2 (0.82)	92.1 (1.88)	94.2 (1.04)
	70	41.7 (3.78)	40.9 (4.01)	41.3 (2.74)
	95	8.2 (1.09)	10.8 (1.72)	9.5 (1.02)
	120	7.6 (1.15)	3.5 (0.63)	5.6 (0.68)
	145	5.3 (0.83)	4.1 (0.81)	4.7 (0.58)
	170	4.3 (0.69)	2.3 (0.58)	3.3 (0.46)
Mandarin Chinese	20	95.7 (1.05)	89.9 (2.12)	93.0 (1.18)
	45	94.1 (1.06)	86.2 (3.17)	90.4 (1.63)
	70	49.3 (6.50)	32.6 (5.97)	41.3 (4.52)
	95	9.6 (2.24)	5.5 (1.69)	7.5 (1.41)
	120	6.1 (2.49)	3.5 (1.22)	4.8 (1.38)
	145	5.1 (2.11)	4.2 (1.17)	4.7 (1.20)
	170	2.1 (0.85)	1.2 (0.69)	1.7 (0.55)

Supplementary Table S2. The process of compensating for the speech rate of a specific talker or a group of talkers. The utterance durations were based on measurements of 35 sentences in American English, German, and Japanese, and those of 18 sentences in Mandarin Chinese. The last column shows the ratio of the duration for a given talker (or pair of talkers) to the average utterance duration in the database.

Language	Duration averaged over all talkers (s)	Talker	Duration (s)	Ratio
American English	2.41	Female	3.11	1.29
		Male	2.47	1.02
		Both talkers	2.79	1.16
German	1.83	Female	2.01	1.10
		Male	2.08	1.14
		Both talkers	2.05	1.12
Japanese	2.64	Female	2.52	0.95
		Male	2.44	0.92
		Both talkers	2.48	0.94
Mandarin Chinese	2.31	Female	2.34	1.01
		Male	2.41	1.04
		Both talkers	2.37	1.03

Supplementary Table S3. Main experiment: Percentage of tone errors observed in Mandarin Chinese. Syllables that were correct in Pinyin spellings but incorrect only in tone signs were counted. Percentages were calculated from a proportion of tone errors to a number of responded syllables in a sentence. Data obtained with 14 participants for the female talker and 13 participants for the male talker. Numbers in parentheses are s.e.m.

Segment duration (ms)	Talker		Average
	Female	Male	
20	3.3 (0.96)	6.8 (1.38)	4.9 (0.84)
45	4.8 (1.00)	5.5 (1.45)	5.1 (0.85)
70	3.4 (1.12)	6.7 (2.84)	4.9 (1.42)
95	5.1 (2.53)	1.3 (0.89)	3.2 (1.36)
120	5.3 (3.09)	1.2 (0.83)	3.0 (1.46)
145	4.8 (2.80)	1.4 (0.79)	2.9 (1.30)
170	2.7 (1.52)	6.4 (2.60)	4.7 (1.56)

Supplementary Table S4. Cross-language experiment: Percentage of tone errors observed in Mandarin Chinese. Syllables that were correct in Pinyin spellings but incorrect only in tone signs were counted. Percentages were calculated from a proportion of tone errors to a number of responded syllables in a sentence. Data obtained with one participant for each talker. The data are also included in Supplementary Table S5. Numbers in parentheses are s.e.m.

Segment duration (ms)	Talker		Average
	Female	Male	
20	5.3 (3.44)	4.3 (2.70)	4.8 (2.07)
45	8.0 (3.76)	14.5 (5.52)	11.3 (3.33)
70	13.9 (6.81)	2.5 (2.50)	8.2 (3.91)
95	4.4 (4.44)	6.7 (6.66)	5.6 (3.79)
120	3.3 (3.34)	0.0 (0.00)	1.7 (1.67)
145	2.2 (2.22)	10.0 (10.00)	6.1 (5.00)
170	14.0 (9.80)	0.0 (0.00)	7.0 (5.17)

Supplementary Table S5. Additional Mandarin Chinese experiment: Percentage of tone errors observed in Mandarin Chinese. Syllables that were correct in Pinyin spellings but incorrect only in tone signs were counted. Percentages were calculated from a proportion of tone errors to a number of responded syllables in a sentence. Data obtained with three participants for each talker. Numbers in parentheses are s.e.m.

Segment duration (ms)	Talker		Average
	Female	Male	
20	5.5 (1.95)	7.2 (2.49)	6.5 (1.56)
45	8.6 (2.21)	11.3 (3.94)	9.9 (2.24)
70	6.8 (2.93)	12.0 (9.03)	9.0 (4.10)
95	1.9 (1.85)	5.6 (5.55)	3.1 (2.16)
120	6.6 (5.09)	8.3 (5.45)	7.4 (3.62)
145	2.6 (1.92)	5.6 (5.56)	3.7 (2.35)
170	13.1 (8.27)	0.0 (0.00)	7.4 (4.80)

References

1. NTT-AT. Multi-Lingual Speech Database 2002 (2002).

2. Boersma, P. & Weenink, D. Praat: Doing phonetics by computer [computer program]. version 6.0.21 (2016). Retrieved on 9 November 2016.
